# Antimicrobial stewardship implementation in primary and secondary tier hospitals in India: interim findings from a need assessment study using mixed method design

**DOI:** 10.1038/s41598-024-78111-0

**Published:** 2024-11-14

**Authors:** Falguni Debnath, Rajyasree Ghosh De, Debjit Chakraborty, Agniva Majumdar, Sandip Mukhopadhyay, Munmun Das Sarkar, Taru Singh, Sanjit Kumar Patra, Surangana Saha, Julius Rehman, Dhiraj Roy, Atreyi Chakrabarti, Sulagna Basu, Asish Kumar Mukhopadhyay, Amitabha Mondal, Shyamal Soren, Kalpana Datta, Shantasil Pain, Supreeti Biswas Mondal, Palash Mondal, Kamini Walia, Dipankar Maji, Alok Kumar Deb, Shanta Dutta

**Affiliations:** 1 ICMR-National Institute of Cholera & enteric Diseases, Kolkata, India; 2 Department of Microbiology, School of Tropical Medicine, Kolkata, India; 3 ECD Division, ICMR, New Delhi, India; 4 Department of Microbiology, Bankura Sammilani Medical College and Hospital, Bankura, West Bengal India; 5 Department of Microbiology, Malda Medical College & Hospital, Malda, West Bengal India; 6 Baruipur Subdivisional Hospital, South 24 Parganas, Baruipur, West Bengal India; 7 Department of Health & Family welfare, Government of West Bengal, Kolkata, India; 8 Department of Paediatrics, Calcutta Medical College and Hospital, Calcutta, West Bengal India; 9 Department of Medicine, IPGMER & SSKM Hospital, Kolkata, West Bengal India; 10 Department of Pharmacology, College of Medicine and Sagar Dutta Hospital, Kolkata, West Bengal India; 11 Integrated Disease Surveillance Program, Government of West Bengal, Kolkata, India; 12 Division of Epidemiology, ICMR -National Institute of Cholera and Enteric Diseases, P-33, CIT Road, Scheme-XM, Beliaghata, Kolkata, 700010 India

**Keywords:** AMR, AMSP, Primary, Secondary, Common infection, APR, Health care, Medical research

## Abstract

Anti-microbial stewardship program (AMSP) is practiced only in tertiary hospitals in India, though, the lower tier hospitals remain the first point of contact in patient care. This study was conducted in lower tier hospitals to calculate antibiotic and multiple antibiotic prescription rate (APR, MPR) for common infections and finding existing strength of health system for optimizing antibiotic prescription. We conducted a cross sectional convergent parallel mix-method study in eight lower tier hospitals of three districts of West Bengal, India. Six hundred OPD prescriptions of UTI, ARI, AUFI, ADD were evaluated. Qualitative data collected through in-depth interviews of medical officers/officers in administrative positions, infection control nurses were analyzed using content analysis method. APR was 63.8% in primary tier hospitals and 60.8% in secondary tier hospitals. The MPR was higher in secondary tier hospital (23.8%). Presence of infection control committee, designated nursing staff, initiation of prescription audit, increased monitoring were identified as few facilitators for future implementation of AMSP in lower tier hospitals. The routine infection control activities of lower tier hospitals are currently delinked from AMR containment measures and thus, customized AMSP needs to be established in these hospitals catering two third of the population of India.

## Introduction

Antimicrobial resistance (AMR) is the most important public health concern of this era, with emergence of highly resistant pathogens causing infections which are not only more difficult to treat but also result in significant increase in morbidity, mortality, healthcare cost^1^.

India has some of the highest AMR rates amongst bacteria that commonly cause infections in both community and healthcare facilities^2^. Moreover, India was the highest consumer of antimicrobials, followed by China and the United states in 2010^3^. The most commonly consumed antimicrobials include broad spectrum penicillin, cephalosporin, fluoroquinolones, macrolides^4^. The amount of cephalosporin consumed nationally increased at an alarming rate with data showing a fourfold increase in consumption rates between 2000 and 2015 .These trends have been observed from studies across the nation^5–7^. Antimicrobials of last choice like Polymyxin, Carbapenems, which are usually reserved for multidrug resistant infections, have also seen a steep increase in consumption rates. All these data reflect the explosive nature of the AMR problem in India. Resistance to commonly used antimicrobials, however cannot be solely ascribed to drug consumption patterns as it is multifactorial^8,9^.

The World Health Organization (WHO) recently published AMR surveillance data from 169 member countries. The report mentioned the lack of national surveillance data on resistant pathogens in India and 14 other member countries^10^.

It is essential to address the AMR challenges holistically with a long-term plan approaching the problem using multidisciplinary strategies. The National Centre for Disease Control, under the Director General of Health Services, Ministry of Health and Family Welfare, Government of India, published The National Policy for Containment of Antimicrobial Resistance, India in 2011. In the year 2017, Government of India prioritized AMR and launched a National Action Plan (2017–2021) in April 2017. A second phase of this has become due in 2022. Indian Council of Medical research (ICMR) also published generic guidelines on antimicrobial stewardship program (AMSP) for India. The purpose of this antimicrobial stewardship Program (AMSP) is to promote rational use of antimicrobials. Few studies done in central India reported baseline inappropriate antimicrobial use in-between 30 and 50%^3,11^, however, reports from other part of the country are lacking and that hinders the discussion on ways to stop irrational antimicrobial prescription. In Indian public health care settings, medical records are poorly maintained, leading to underestimation and misclassification of the underlying etiology associated with the prescription of antimicrobials. ICMR-NICED also conducted a study on Rational Use of Medicine at tertiary level and identified deficiencies in current antimicrobial prescription practices for new outpatient cases of diarrhoea and Acute Respiratory Infection (ARI). It was observed that despite having many available guidelines (NCDC, ICMR, WHO, State) on antimicrobial use in India, hardly any prescriber followed it^12^. Currently, AMR surveillance in India is tertiary hospital based and concentrated on characterization of resistant isolates only^13^. Hence, most of the published document on containment of AMR are based on findings from tertiary care centers and that may be not applicable to lower tier hospitals. As per ICMR AMSP guidelines each healthcare setting must have an identified stewardship team, hospital specific antimicrobial policy, guidelines for antimicrobial treatment and prophylaxis, periodical training on AMSP, monitoring and reporting of antimicrobial use. A study done in a tertiary care hospital in Ujjain reported a 20% increment in appropriateness of base line antimicrobial prescription following adapting AMSP in one of the units. The study also demonstrated significant decrease in mean hospital stay duration in the intervention unit^11^. So far, we only have reports from limited central Indian tertiary care settings where AMSP has been implemented in project mode to document its effectiveness, reports from other parts of the country and lower tier hospitals are lacking. However, 70% of Indian population is still rural and they receive services from primary and secondary tier hospitals mostly and data on AMR from those set ups in India is almost absent. Hence, in this paper we reported the antimicrobial prescription pattern for common illnesses like: presumptive urinary tract infection (UTI), acute respiratory tract infection (ARI), acute undifferentiated febrile illness (AUFI), acute diarrhoeal diseases (ADD) in primary and secondary tier hospitals and how this may be optimized with the available limited resources.

### Objectives

1. To calculate antimicrobial prescription rate (APR), multiple antimicrobial prescription rate (MPR) for common infections in primary and secondary tier hospitals.
2. To calculate the proportion of matching of empirical treatment with antibiogram of isolated organisms from common illnesses in primary and secondary tier hospitals.
3. To understand the existing scope of health system for optimizing use of antimicrobials for common infections.

## Methods

### Study design

This was a Multitier facility based convergent parallel study using cross-sectional design. Here, we reported initial findings of the study, conducted during November 2022- December 2023.

### Study area

In India, we have three tiers of hospitals in Government system, namely Primary; Secondary and Tertiary tier hospitals which are mostly teaching hospitals. This study was conducted in primary and secondary tier hospitals of three districts in West Bengal province of India. In this paper we reported findings of the study from five primary and three secondary level hospitals of South 24 Parganas district, Bankura district, Malda district. These districts are geographically distinct and have different population composition.

### Study population

To answer the first two objectives, we studied the prescriptions of patients with common infection namely: Acute Respiratory Infection (ARI); Urinary Tract Infection (UTI), Acute Diarrhoeal Disease (ADD), Acute Undifferentiated Febrile Illness (AUFI). Appropriate biological specimens were also collected from them as a part of the study.

To meet the last objective, we collected qualitative data from medical officers, medical officers in administrative position, infection control nurses.

#### Sample size

We examined 600 prescriptions from outpatient departments as per WHO guideline^13^. Data on Prescriptions were collected through consecutive sampling on randomly selected days from different clinical disciplines such as Medicine, Paediatrics, General Outpatient Dept (OPD).

We collected qualitative data through eight in-depth interviews from required members.

### Operational definitions

#### ARI

Both upper respiratory tract infections (URIs) or lower respiratory tract infections (LRIs) were considered in this study. The URIs included conditions like rhinitis (common cold), sinusitis, ear infections, acute pharyngitis or tonsillopharyngitis, epiglottitis, and laryngitis. The LRIs included conditions like pneumonia and bronchiolitis^14^.

#### ADD

ADD was defined as the passage of three or more loose or liquid stools per day (or more frequent passage than is normal for the individual). Frequent passing of formed stools was not considered as diarrhoea, nor was the passing of loose, “pasty” stools by breastfed babies^15^.

#### AUFI

AUFI was defined as patients having fever of < 14 days duration without any evidence of organ or system specific aetiology^16^.

#### Presumptive UTI

The following patients were considered as presumptive UTI cases:

Acute dysuria alone, OR Fever (> 37.9°Cor 1.5 °C increase above baseline temperature) and at least 1 of the following: New or Worsening (a) Urgency, (b) Frequency (c) Suprapubic pain, (d) Gross haematuria, (e) Costovertebral tenderness and (f) Urinary incontinence^17^.

### Sample collection and processing

Appropriate biological specimens were collected from Out-Patients Departments (OPDs) following standard aseptic procedures and then transported to the laboratories for further processing under strict temperature control.

Urine, stool/rectal swab, throat swab were collected from presumptive UTI, ADD and ARI cases respectively. Clean catch mid-stream urine sample and stool were collected in sterile containers (Sterile Multipurpose Clinical Sample Collector; HIMEDIA PW1179), rectal swab was collected in Cary Blair Transport Medium (prepared in-house) and throat swab was collected in Amies Medium (HIMEDIA MS684B). Sample processing and identification of microorganisms were done by conventional methods^18^.

For primary plating and culture, MacConkey agar and CLED agar (Cystine, lactose, electrolyte-deficient (CLED) medium) were used for urine sample, MacConkey agar and blood agar medium for throat swab culture, and MacConkey agar, HEA (Hektoen Enteric Agar), TCBS (Thiosulfate Citrate Bile Sucrose) agar medium for stool or rectal swab. Colony count ≥ 10^5^was reported as significant for urine sample. If any previous history of antimicrobial consumption was present, then colony count 10^3^ was considered as significant. Gram staining and microscopy were performed from isolated colonies, and then were further subjected to suitable biochemical tests for identification^19^. If more than two organisms were isolated from a urine specimen, it was considered contaminated and rejected.

After isolation of the organisms, they were tested for antimicrobial sensitivity by Kirby Bauer disc diffusion methods, following the Clinical Laboratory Standards Institute (CLSI) guidelines and were reported as “sensitive’ or “resistant”.

### Data collection

After obtaining written informed consent/assent from patient/caregiver/party, photo of the prescriptions was clicked hiding the identifier and appropriate specimens were also collected. Before qualitative data collection, we obtained written informed consent from the interviewees.

### Ethical approval

Institutional Ethics Committee approval was obtained from ICMR-NICED, Kolkata (IEC NO: A-1/2021/IEC) following adherence to all the methods as laid down in ICMR Ethical Guidelines for Biomedical Research 2017 and administrative approval was obtained from West Bengal State Health Department.

### Data analysis

We have used the guidelines to be used for primary and secondary tier hospitals. These guidelines are meant for Primary and Secondary care public health facilities providing Out-Patient care at District Hospitals (DHs), Sub-divisional Hospitals (SDHs), Community Health Centres (CHCs) and Primary Health Centres (PHCs)^20,21^.

Series of meetings were conducted with pediatricians, clinicians, pharmacologists to build upon the OPD prescription evaluation guideline issued by the state health department of West Bengal^22^. We devised the criteria of evaluation based on that.

Standard indicators in line with core Indicators of WHO were calculated out of the photographed prescriptions^12,21^.

Qualitative data were analyzed obtaining manual content analysis method and presented the information through abstraction matrix. IDIs were conducted till the answers got saturated.

## Results

### General findings of prescription evaluation

A total number of 600 prescriptions were evaluated and 511 clinical samples of patients with common infections from outdoor (OPD) were collected and subjected to microbiological testing.

Among the 600 prescriptions from OPD, 268 (44.7%) were from primary tier and 332 (55.3%) were from secondary tier hospitals. Of the 600 evaluated prescriptions, 309 (51.5%), 186 (31%), 75 (12.5%) and 30 (5.0%) were of UTI, ARI, AUFI and ADD respectively. More than 80% of the evaluated prescription had signature of the prescriber, however History of previous medication, was present in 2.1% (7/ 268) prescription of secondary tier (Table 1). Among the prescription of primary tier, history of co-morbidities was mentioned in 7.8% (21/ 332) and the value was almost similar to that of secondary tier (Table 1).

**Table 1:** Findings of OPD prescription evaluation of common illnesses from primary and secondary tier hospitals; West Bengal; India; 2023.

Indicator used	Primary level (*N* = 268)	Secondary level (*N* = 332)	X^2^ Value (*P* value)
	n (%)	n (%)	
Signature present	224 (83.5)	282 (84.9)	0.21 (0.64)
Medication History present	12 (4.5)	7(2.1)	2.7 (0.09)
History of comorbidity present	21(7.8)	20(6)	0.76 (0.38)
Use of non-standard abbreviation	90 (33.6)	92 (27.7)	2.4 (0.12)
Complete generic prescription	54 (20)	32 (9.6)	11.7 (0.0006)
Average number of drugs prescribed per prescription	3.85	2.8	**-**

### Antimicrobial prescription related indicators from the evaluated prescriptions

The APR of primary and secondary tier hospitals for common infections was 63.8% (171) and 60.5% (201) respectively. The MPR was 9.4% and 23.8% respectively in primary and secondary tier hospitals.

Among the evaluated prescriptions, we found that only in 15 out of 171 (8.8%) in primary tier and 16 out of 201 (7.9%) prescriptions from secondary tier, prescriber prescribed the antimicrobial as per available guideline (Table 2). In the primary care hospitals, out of 171, in 89 (52%) prescriptions antimicrobials from Access group (WHO-AWaRe) were prescribed and in 90 (52.6%) prescriptions, antimicrobials from Watch group (WHO-AWaRe) was prescribed. However, in very a smaller number of prescriptions, the prescribed empiric antimicrobial matched with the respective antibiogram report (Table 2).

**Table 2:** Empiric antimicrobial prescription indicators, OPD prescription evaluation of common illnesses from primary and secondary tier hospitals; West Bengal; India; 2023.

Indicator used	Primary level	Secondary level	X^2^ Value (*P* value)
Empiric antimicrobial matched the C/S report	2/66 (3%)	11/113 (9.7%)	2.7 (0.09)
Antimicrobials prescribed according to treatment guideline	15/171 (8.8%)	16/201 (7.9%)	0.07 (0.77)
Antimicrobials from Access Group (WHO- AWaRe)	89/171 (52%)	106/201 (52.7%)	0.017 (0.89)
Antimicrobials from Watch Group (WHO- AWaRe)	90/171 (52.6%)	130/201 (64.7%)	5.5 (0.01)
APR%	171/268 (63.8%)	201/332 (60.5%)	0.67 (0.41)
MPR%	16/171 (9.4%)	48/201(23.8%)	13.6 (0.0002)

### Results of microbiological testing of collected specimens

Out of the 600 evaluated OPD prescriptions, specimens were collected from 511 patients. Among them, 33% (169/511) specimens were collected from primary tier hospitals. Out of the 342 specimens collected from secondary tier hospital, more than one third was urine specimen collected from presumptive UTI cases and 16% (57/342) was stool/rectal swab collected from ADD patients (Table 3). Rate of culture isolation remained almost same in both primary and secondary tier hospitals (Table 3).

**Table 3:** Distribution of culture positivity rate among collected clinical samples across primary and secondary tier hospitals, West Bengal, 2023.

Treatment care	Urine	Culture positivity rate	Stool/Rectal swab	Culture positivity rate	Throat swab	Culture positivity rate
Primary care	120	16 (13.3%)	38	08 (21.0%)	11	05 (45.4%)
Secondary care	268	28 (10.4%)	57	09 (15.8%)	17	05 (29.4%)
Total	388	44 (11.3%)	95	17 (17.9%)	28	10 (35.7%)

*Escherichia. coli* remained the major organism causing UTI in both primary and secondary tier hospitals (Table 4). However, *Klebsiella spp.* was identified more from urine specimens collected from secondary tier hospitals. *Shigella flexneri 2a* was identified to be the major organism causing acute diarrhoeal diseases both in primary and secondary tier hospitals. *Staphylococcus spp.* infection was found more in secondary tier hospitals (Table 4).

**Table 4:** The distribution of identified bacterial pathogens from different clinical samples across primary and secondary tier hospitals, West Bengal, 2023.

Types of specimen & isolated organisms	Primary care	Secondary care
Urine (*N* = 44)	*N* = 16	*N* = 28
Gram + ve		
*Enterococcus faecalis* (*N* = 03)	02 (12.5%)	01 (3.6%)
*Staphylococcus aureus.* (*N* = 02)	01 (6.25%)	01 (3.6%)
*Micrococcus spp.* (01)	00	01 (3.6%)
Gram -ve		
*Escherichia coli* (*N* = 30)	11 (68.75%)	19 (67.9%)
*Klebsiella spp*. (*N* = 06)	01 (6.25%)	05 (17.9%)
*Citrobacter freundii* (*N* = 01)	01 (6.25%)	00
*Aeromonas hydrophila* (*N* = 01)	00	01 (3.6%)

Stool/Rectal swab (*N* = 17)	*N* = 08	*N* = 09
Gram -ve		
*Shigella spp.*(*N* = 13)	05 (62.5%)	08 (88.9%)
*Salmonella spp*.(*N* = 03)	02 (25%)	01 (11.1%)
*Vibrio cholera OI ogawa* (*N* = 01)	01 (12.5%)	00

Throat swab (*N* = 10)	*N* = 05	*N* = 05
Gram + ve		
*Enterococcus faecalis* (*N* = 01)	01 (20%)	00
*Staphylococcus spp.* (*N* = 06)	02 (40%)	04(80%)
Gram -ve		
*Klebsiella spp.* (*N* = 03)	02 (40%)	01(20%)

### Antimicrobial resistance profile of major organisms identified from urine specimen, rectal swab and throat swab

**Fig. 1:**
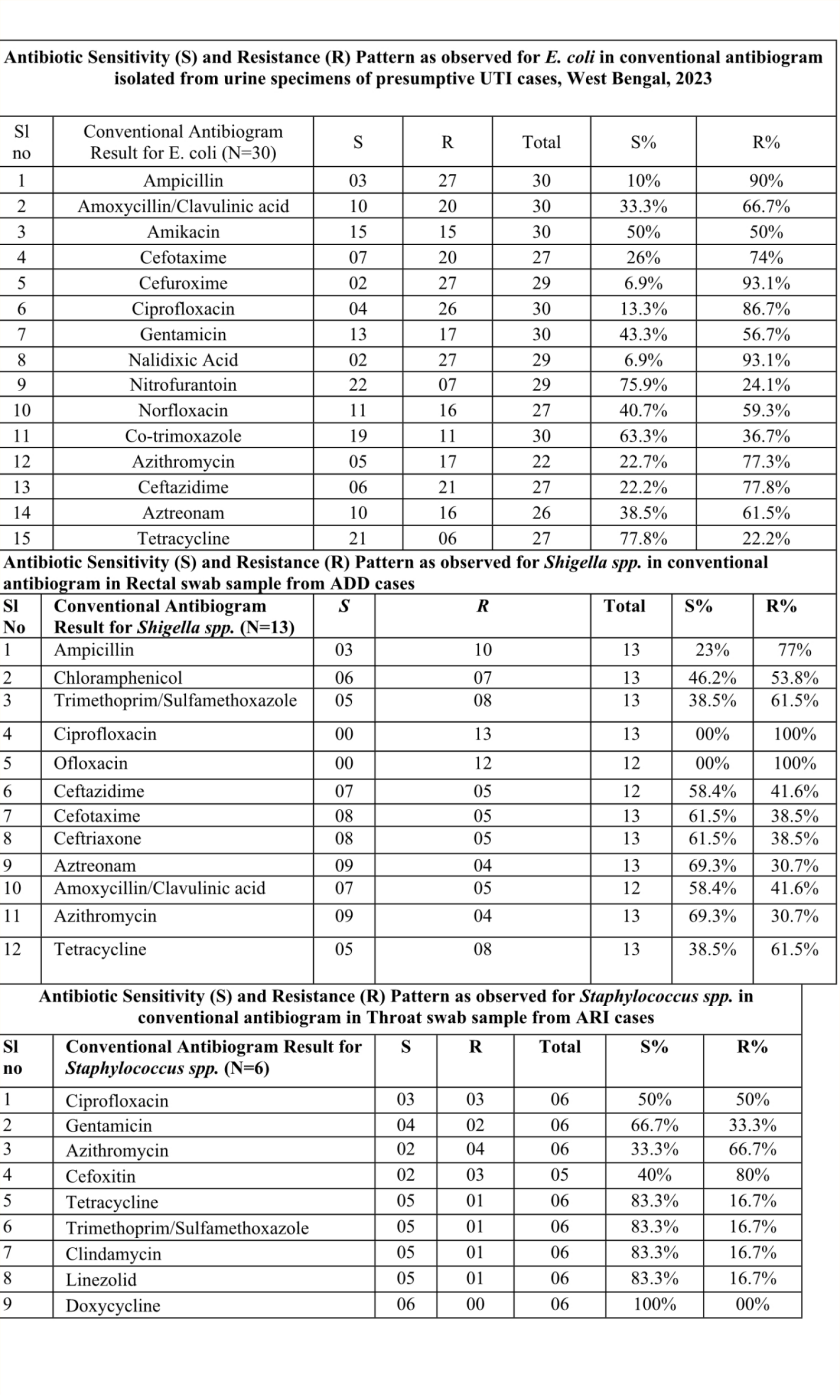
Antibiogram of major organisms identified from presumptive UTI; ADD, ARI cases, West Bengal, 2023.

The overall AMR profile of isolates is presented in Fig. 1. In this study, *E coli* in urine sample was mostly resistance to Cefuroxime and Nalidixic Acid (93.3%) followed by Cephalexin (88.9%), Ampicillin (86.7%) and Ciprofloxacin (83.9%). Nitrofurantoin and tetracycline were sensitive (Fig. 1).

*Shigella flexneri* was the most common isolated organism from rectal swabs collected from ADD across tiers. In both the tiers, it was completely resistant to fluroquinolones which are been supplied to hospitals from Government, though they exhibited fair sensitivity to azithromycin, a drug from macrolide group (Fig. 1). Trimethoprim/Sulfamethoxazole identified to be a sensitive drug against Staphylococcal infection causing ARI (Fig. 1).

### Identifying existing scopes in health system for optimizing use of antimicrobials for common infections

**Fig. 2:**
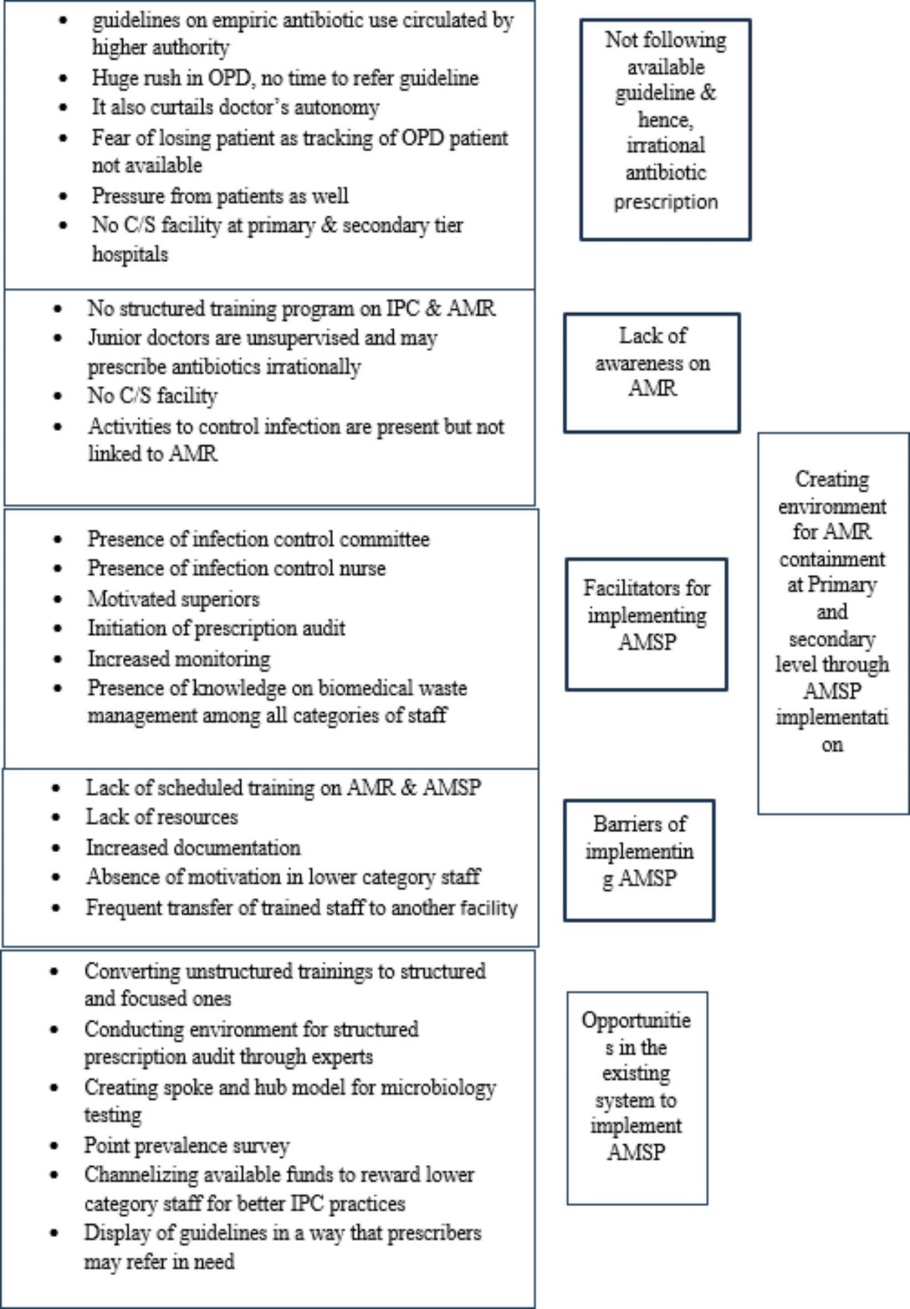
Identified scopes for intervention to optimize rational antimicrobial use across primary and secondary tier hospitals, West Bengal, 2023.

Through six in-depth interviews, it was identified that despite availability of guidelines on empiric prescription of antimicrobials for common illnesses, use of those guidelines in day-to-day practice was absent and hence, over use of antimicrobials remained to be a problem. One of the medical officers in administrative position mentioned, “*Doctors are very busy in OPD*,* it is not possible to go back to guidelines always and doctors have the fear also that OPD patients cannot be tracked*,* so to be in safe side*,* doctors prefer to give antimicrobial during the first visit*”. Lack of awareness on AMR both among clinicians and nurses emerged to be another major theme. One of the interviewees mentioned that “*We do not have any specific training on AMR or the current trends in it and again absence of C/S facility at primary and secondary level imposes barrier in having current knowledge on AMR”.* Introduction of a position of infection control nurse at all tiers of hospitals, distribution of guidelines on empiric treatment for common infections, introduction of prescription audit using hospitals’ own staff, increased monitoring by district health authorities identified to be the facilitators for implementing antimicrobial stewardship program at primary and secondary level. Converting unstructured trainings in to scheduled focused training programs, channelizing available hospital funds for motivating nursing staff, lower category staff for better infection control practices; structured prescription evaluation through experts; creating spoke and hub model for microbiological testing; creating structure for point prevalence survey at local level were identified as the opportunities in the system for implementing AMSP to optimize antimicrobial use and contain AMR in long run (Fig. 2).

## Discussion

High APR was observed in both primary and secondary tier hospitals in common infections, however MPR was much higher in secondary tier hospitals. The secondary tier hospitals in India have specialized OPDs however, they do not have microbiological testing facility. The primary hospitals have only general OPD. In this study, we observed almost similar isolation rates from clinical specimens collected from UTI, ARI and ADD patients attending OPDs of primary and secondary tier hospitals.

The observed APR in primary and secondary hospitals was as high as this was observed in tertiary care centers of West Bengal^23^. Higher APR in tertiary tier hospital may be described in terms of getting more referral cases or critical cases even in OPDs. However, primary and secondary tier hospitals are usually the first point of contact with a formal clinician in Indian setting and high APR in such condition highlights the need of immediate containment measures. Moreover, a very smaller number of these empiric antimicrobials matched with respective antibiogram report. In absence of any microbiological facility, point prevalence survey data, unstructured prescription audit, rampant prescription of antimicrobials in common infection posses’ threat on existing containment measures of AMR in India.

The prescribers in both the tiers prescribed almost similar proportion of antimicrobials from “access” and “watch” category. The WHO classified the antimicrobials in access, watch and reserve group with an aim to rationalize its prescription. The WHO has also mentioned that countries to set a target of ≥ 60% of total antimicrobial consumption being from Access group antimicrobials^24^. In this present study, it was far behind in both the tiers. However, according to Phrmatrack data, watch group antimicrobials are mostly abused in Indian market^25^. Pharm track data is a reflection of pan India sale data of antimicrobials, however, in Indian Government run hospitals, patients are also supplied medicines free of cost. We also observed that in primary tier hospitals access to watch ratio was 0.9 and in secondary tier hospitals it was 0.8 which was below from 1.5 in both the setups. This finding was similar to another study conducted in Tamilnadu, where they reported it to be 0.7^26^. The lower access to watch ratio in antimicrobial prescription indicates it as an emerging public health concern and need for having surveillance on antimicrobial prescription and dispensing in Government hospitals.

In the private sector, increase in consumption of watch category antimicrobial may be defined by :1. Increased affordability, 2. Surplus supply in local pharmaceutical market^27^. However, in Government run set ups, increased use of watch category drugs not only imposes policy level challenges but also indicates towards augmentation in antimicrobial stewardship program implementation at those levels which are currently absent. Moreover, almost in each studied common conditions, we observed similar isolation rates in conventional culture and also observed that not even 10% of the cases, empiric treatment matched antibiogram result. In the current health care delivery system in India, microbiological testing facility is only present in tertiary care centers^28^. Even, in current system there is no provision of having any surveillance over organisms causing diseases and their antibiogram in lower tier hospitals. Lack of resources, rush in OPDs, absence of mechanism to track OPD patients further complicates the situation. Studies done in Asian countries also highlights limited knowledge of physicians in AMR^27,28^. In absence of any structured training program on AMR and AMSP, we observed that physicians of primary and secondary tier hospitals were disconnected with the facts on magnitude of AMR in India. Different studies conducted across the globe have identified structured training as one of the tools within the framework of AMSP to rationalize antimicrobial prescription^29^. Even they have identified the need of alleviating concerns over diagnostic uncertainties^29^. We have observed similar findings in the present study. Currently AMSP is only practiced in limited tertiary care centers in India where diagnostic facilities are available, but the lower tier hospitals cater to patients with infection in large numbers but do not have the sufficient diagnostic facilities^28^. However, currently, all the government run hospitals have one infection control nurse for supervising the infection prevention and control activities of the hospital which may be a starting point to link the IPC activities with AMR containment measures^30^. The lower tier hospitals need to be empowered to have their own AMSP in place to participate in the national drive of AMR containment. Studies done to reduce inappropriate antimicrobial prescribing for childhood (2–14years old) upper respiratory tract infections (URTIs) in rural hospitals in China found that with education, training and review of prescription there was a long-term reduction in inappropriate antimicrobial prescription^31^. However, research studies have their own limitation in terms of being in place for shorter duration and hence, the seasonal fluctuation in prescribing pattern could not be analysed. AMSP is a globally identified tool for AMR containment and the Indian lower tier hospitals have all the possible reasons to have their AMSP in place.

## Data Availability

Data related to the study are available with the first author and the corresponding author.
